# Optical coherence tomography for label-free detection and characterization of methicillin-resistant *S. aureus* biofilms

**DOI:** 10.1117/1.JBO.30.4.046003

**Published:** 2025-04-01

**Authors:** Natalia Demidova, Jason R. Gunn, Ida Leah Gitajn, Ilya Alex Vitkin, Jonathan Thomas Elliott, Valentin V. Demidov

**Affiliations:** a Dartmouth Health, Department of Orthopaedics, Lebanon, New Hampshire, United States; b University of Toronto, Department of Medical Biophysics, Toronto, Ontario, Canada; c Dartmouth College, Geisel School of Medicine, Hanover, New Hampshire, United States; d University of Toronto, Department of Radiation Oncology, Toronto, Ontario, Canada; e Dartmouth College, Thayer School of Engineering, Hanover, New Hampshire, United States

**Keywords:** optical coherence tomography, infection, biofilm, texture analysis, speckle statistics

## Abstract

**Significance:**

Orthopedic implant-associated infections cause serious complications primarily attributed to bacterial biofilm formation and are often characterized by increased antibiotic resistance and diminished treatment response. Yet, no methods currently exist to identify biofilms intraoperatively—surgeons rely solely on their eyes and hands and cannot detect or differentiate infected tissue to determine the location and extent of contamination.

**Aim:**

As the first step in addressing this unmet clinical need, here, we develop an optical coherence tomography (OCT)-based imaging method capable of detection *in situ* and quantification of one of the most dangerous orthopedic biofilms formed by methicillin-resistant *Staphylococcus aureus* (MRSA).

**Approach:**

Growing biofilms on orthopedic hardware, we identify MRSA distinct optical signature through histogram-based multi-parametric texture analysis of OCT images and support the findings with bioluminescence imaging and scanning electron microscopy. Under identical experimental conditions, we identify an optical signature of *Escherichia coli* (*E. coli*) biofilms and use it to distinguish and quantify both species within MRSA–*E. coli* biofilms.

**Results:**

The developed OCT-based methodology was successfully tested for (1) MRSA colonies delineation, (2) detection of metal hardware (an important feature for clinical translation where the metal surface of most orthopedic hardware is not flat), (3) automated quantification of biofilm thickness and roughness, and (4) identification of pores and, therefore, ability to evaluate the role of porosity—one of the critical biological metrics in relation to biofilm maturity and response to treatment. For the first time, we demonstrated complex pore structures of thick (>100  microns) MRSA biofilms *in situ* with an unprecedented level of detail.

**Conclusions:**

The proposed rapid noninvasive detection/quantification of MRSA biofilms on metal surfaces and delineation of their complex network of pores opens new venues for label-free MRSA detection in preclinical models of trauma surgery, expansion to other bacterial strains, and further clinical translation.

## 1 Introduction

According to the latest estimation by the US Center for Disease Control (CDC), more than two million people are sickened every year in the United States with antibiotic-resistant infections, with tens of thousands dying as a result.^1^ In orthopedic trauma surgery, infection leads to nearly half of unplanned surgical procedures, prolonged morbidity and potential limb loss with economic cost of more than $500 million per year.^2^ Methicillin-resistant *Staphylococcus aureus* (MRSA) is one of the leading causes of these serious surgical complications and deaths in patients, classified by CDC as an urgent threat to the US population. Life-threatening MRSA biofilms form on bones, soft tissues, and implants and are extremely hard to eradicate;^3^ therefore, tissues and implants with biofilm must be removed.^4^

However, there are currently no intraoperative tools to visualize these biofilms to guide resection. Surgeons rely solely on their eyes and hands while in the operating room and cannot detect or differentiate infected regions to assess the best course of treatment.^5^ Over-debridement results in soft tissue and/or bone defects with increasingly challenging reconstructive needs, whereas under-debridement and infected implant oversight results in persistent biofilm and risk for infectious recurrence.

To confirm the presence of MRSA infection, the standard-of-care is random sampling of the surgical site for microbiology culture in 1 to 3 days, with a high risk of missing the infection site. It does not provide real-time, actionable information to the surgeon and lacks information about the location, thickness, or density of biofilm. An intraoperative technique capable of providing feedback about the local extent of bioburden would enable surgeons to make better-informed decisions regarding therapeutic strategies to control infection, such as deciding between retention and explantation of hardware and/or extent of debridement.

There are several advanced imaging and sensing modalities routinely used for laboratory-based biofilm imaging and characterization, including scanning electron microscopy,^6^ helium ion microscopy,^7^ enhanced Raman spectroscopy,^8^ confocal scanning laser microscopy,^9^ light-sheet microscopy,^10^ spinning disc systems,^11^ X-ray computed microtomography,^12^ and magnetic resonance microscopy.^13^ Violet-light excitation fluorescence imaging has demonstrated the potential for bedside visualization of bacterial presence in infected wounds.^14^ Appearing particularly promising for intraoperative use is optical coherence tomography (OCT)^15^—a noninvasive real-time imaging modality capable of visualizing biofilm morphology.^16^ Often called a “virtual” optical biopsy, OCT offers nonionizing depth-resolved label-free functional imaging of tissues *in vivo* at resolutions approaching optical microscopy.^17^ Unlike electron microscopy or confocal microscopy, OCT does not require sample preparation or fixation, allowing for live, real-time imaging of biofilm development without the need for contrast agents. This makes OCT particularly well-suited for monitoring biofilm formation on medical implants during clinical procedures.

In previous studies, we and others showed that OCT can differentiate between healthy and cancer cells,^18^^–^^20^ is sensitive to apoptotic and dead cells,^21^^,^^22^ and can visualize orthopedic biofilms.^23^^,^^24^ A recent investigation demonstrated that OCT has a potential for differentiation between *H. influenza*, *S. pneumonia*, *M. catarrhalis*, and *P. aeruginosa* bacterial strains by analyzing OCT signal optical properties.^25^ Thus, positing that MRSA has a distinct optical signature, here, we present a methodology for its detection in laboratory-grown orthopedic biofilms, focusing on implant-occurring biofilms and performing histogram-based multi-parametric texture analysis of their OCT images. Using a bioluminescent MRSA strain and scanning electron microscopy to guide OCT methodology development, we identify porous biofilm structures and delineate biomass from metal hardware surfaces for further color-coded visualization, analysis, and quantification. In addition, we perform the same texture analysis to identify the optical signature of *Escherichia coli* (*E. coli*) and apply it to differentiate between the two bacteria in dual-species MRSA–*E. coli* biofilms.

## 2 Materials and Methods

### 2.1 MRSA and *E. coli* Biofilm Growth Model

**Fig. 1:**
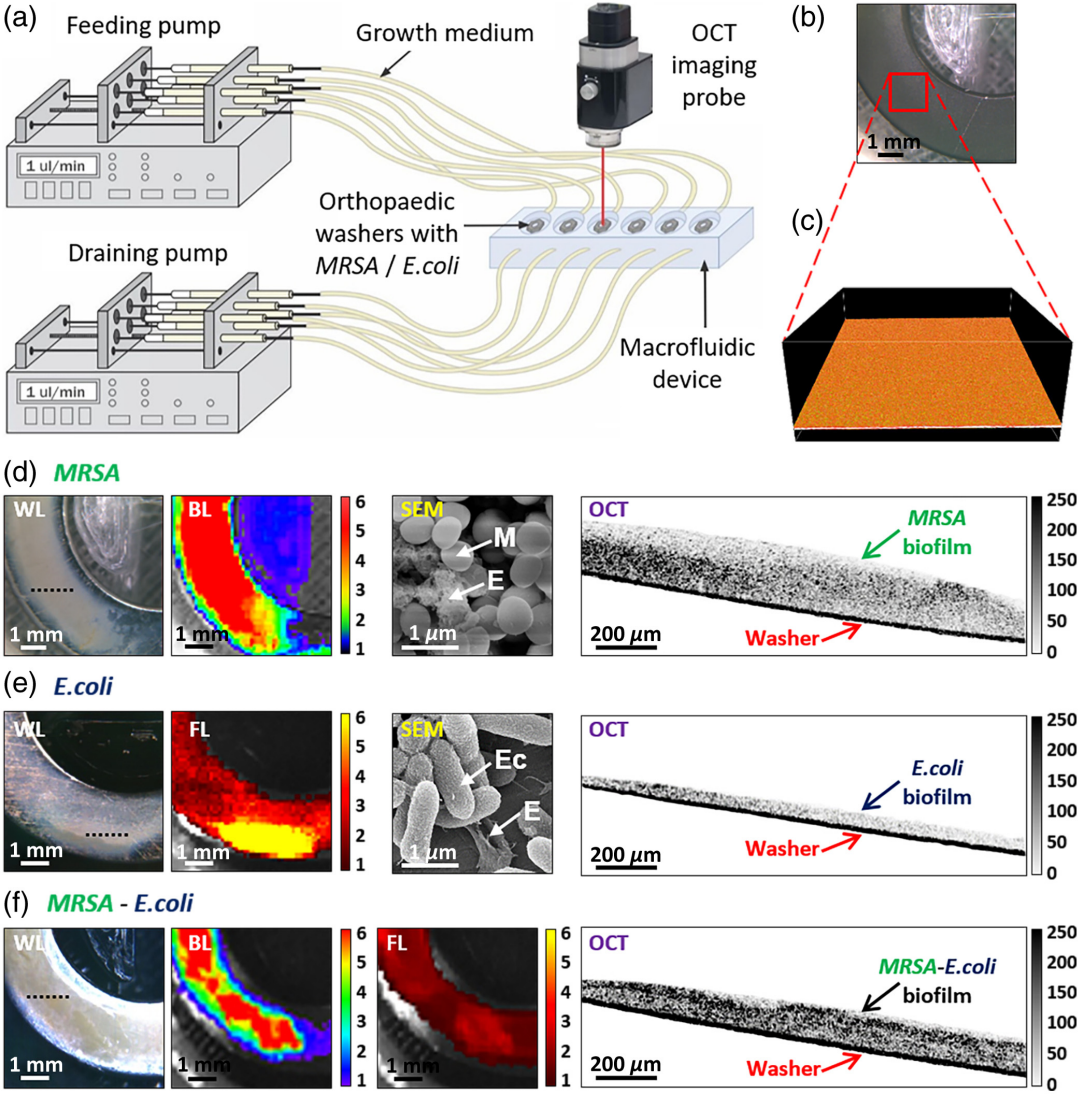
(a) Schematic of a macrofluidic model of orthopedic hardware biofilm growth setup. (b) Negative control example—microphotograph of a titanium washer held for 72 h in MRSA-free broth, and corresponding (c) 3D-rendered OCT image of its surface from the $1.5\times 1.5\;{\mathrm{mm}}^{2}$ area, labeled with a red rectangle in panel (b). (d) Panel of a SS washer white light (WL) microphotograph with MRSA biofilm, bioluminescence (BL, units are in $\times 10^{7}\;p/s/{\mathrm{cm}}^{2}/\mathrm{sr}$), SEM, and cross-sectional OCT images. *M*–MRSA, *E*–extra-cellular polymeric substance (EPS). (e) Panel of an SS washer microphotograph with *E. coli* biofilm, fluorescence (units are in $\times 10^{7}\;p/s/{\mathrm{cm}}^{2}/\mathrm{sr}$), SEM, and cross-sectional OCT images. *Ec*–*E. coli*, *E*–EPS. (f) Panel of an SS washer microphotograph with MRSA*–E. coli* biofilm, MRSA bioluminescence, *E. coli* fluorescence, and cross-sectional OCT images. Black-dotted lines in microphotographs indicate the locations of the corresponding OCT cross-sectional images. OCT image color bars represent signal magnitude (reflectivity, arbitrary units).

A macrofluidic model was developed in-house for *in vitro* growth of bioluminescent strain SAP231^26^ of patient-derived MRSA and fluorescently labeled *Escherichia coli* AR3110 on orthopedic hardware, described in detail elsewhere^27^^,^^28^ and based on established biofilm growth protocols.^29^ Briefly, three titanium and three stainless steel Asnis III Stryker washers (widely used on implants in orthopedic surgery) were situated inside a 3D-printed macrofluidic device schematically shown in Fig. 1(a), connected to feeding and draining syringe pumps (BS-8000, Braintree Scientific, Braintree, Massachusetts, United States) through segments of #30 microtubing (Cole Parmer, Vernon Hills, Illinois, United States). Microtubes were attached to 27 G needles on 1 mL syringes and plumbed into device inlets. Pumps were continuously fed tryptic soy broth with 5% fetal bovine serum and drained each well of the device at 1  μL/min flow rate for 72 h to grow 60- to 180-μm-thick biofilms on washer surfaces.

Fluorescently labeled *E. coli* AR3110 bacterial strain was cultured in 5-mL tryptic soy broth overnight at 37°C at 250 rpm in an orbital shaking incubator, then pelleted, washed, and standardized prior to inoculation into macrofluidic devices. A total of 108 titanium and SS washers were used for experiments to grow biofilms in 18 macrofluidic devices (three for controls, six for MRSA, six for *E. coli*, and three for MRSA and *E. coli*).

### 2.2 Biofilm Imaging

OCT system (Ganymede II, Thorlabs, Newton, New Jersey, United States) was used for taking white-light microphotographs [Fig. 1(b)] with the color camera integrated into the OCT probe and three-dimensional imaging of washer surfaces with or without biofilms on them (as in the TSB-only case with no MRSA present [Fig. 1(c)]). Six $1.5\times 1.5\times 0.6\;mm^{3}$ OCT images were taken systematically across the entire top surface of each washer. The sequential process of imaging at six locations of all six washers in each device led to changes in the angle of incidence, as each washer was adjusted manually under the probe during imaging. In addition, due to the natural variability in biofilm thickness, the focal distance was adjusted to the middle of the biofilm, which in turn changed the relative positioning of both the biofilm and the washer during imaging. Such a small $1.5\times 1.5\;mm^{2}$ field of view was chosen because in clinical applications, particularly in surgical environments where time is often limited, surgeons typically face the challenge of efficiently assessing specific regions of interest, particularly when dealing with complex or large surgical sites. In these scenarios, the ability to focus on localized areas suspected of infection, rather than attempting to image an entire surface at once, provides significant clinical value.

IVIS spectrum (PerkinElmer, Shelton, Connecticut) imaging system subsequently detected MRSA bioluminescence and *E.coli* fluorescence as shown in Figs. 1(d)–1(f). Helios 5CX DualBeam (Thermo Fischer, California, United States) scanning electron microscope (SEM) was used to confirm the presence of MRSA in biofilms, as described in Ref. 27.

### 2.3 OCT Image Analysis for MRSA Colonies, Biofilm Pores, and Hardware Surface Detection

**Fig. 2:**
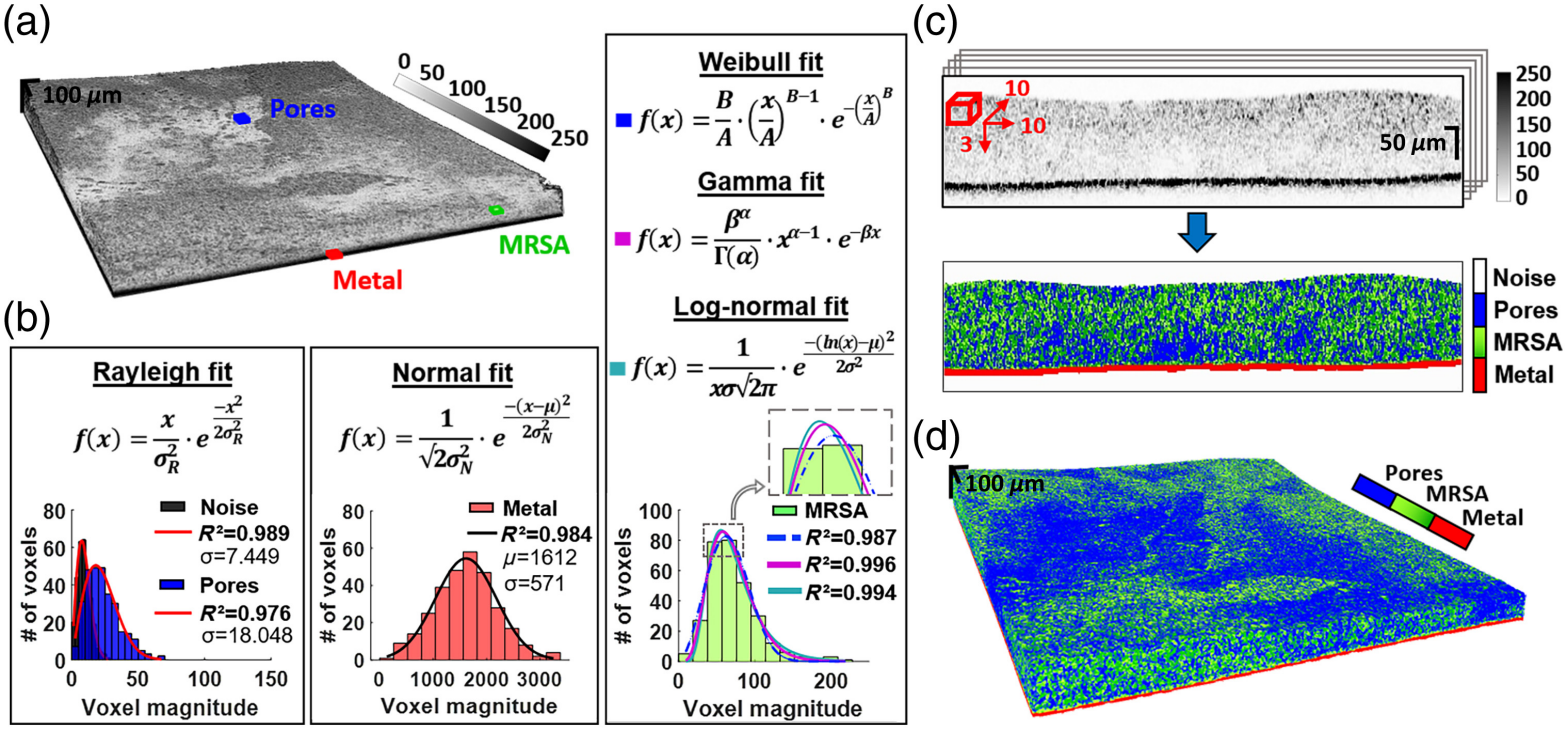
OCT-based biofilm delineation method. (a) 3D OCT image of biofilm ($1.5\times 1.5\;{\mathrm{mm}}^{2}$ field of view) grown on a stainless steel washer; (b) histograms from OCT image regions of noise (black), pores (blue), metal hardware (red), and MRSA (green), and their fitting with Rayleigh, normal, Weibull (W), gamma (G), and log-normal (LN) distributions. An embedded image in the bottom right of the W/G/LN panel (indicated by the arrow) demonstrates the shifts of LN (green) and W (dashed blue) fitting curves relative to the G (magenta) curve to account for small pores and EPS matrix in MRSA biofilm. (c) Schematic representation of 10 consecutive B-scans used in each iteration of the algorithm. Sliding subVOI (red) of 10×10×3  voxels was used for histogram plotting and fitting for the detection of noise (black), pores (blue), metal surface (red), and MRSA (green) shown in the resulting parametric image below; (d) 3D-rendered parametric image of biofilm after texture analysis of all B-scans.

OCT data processing was performed in Matlab (2023a, MathWorks, Natick, MA, United States). Three-dimensional OCT images of biofilms [3000×3000×300  voxels, or $1.5\times 1.5\times 0.6\;mm^{3}$, as shown in Fig. 2(a)] were converted from dB to magnitude and divided into 10×10×3  voxel-sized overlapping sub-volumes (subVOIs) for parametric processing. A novel probability distribution function (PDF) analysis methodology was developed based on spatial OCT speckle statistics, a method increasingly used for biomedical applications.^30^ Signal histogram curves from each subVOI were fit with five statistical PDFs—Rayleigh (R), gamma (G), normal (N), Weibull (W), and log-normal (LN) as shown in [Fig. 2(b)] to estimate fit parameters and goodness-of-fit $R^{2}$, using nonlinear regression (minimization of squared residuals).^31^ Previously, we showed that noise and OCT signals from low-scattering regions are fit well by the Rayleigh distribution and separable in fitting parameter σ and $R^{2}$ space.^32^^,^^33^ Similarly, here, we determined regions of these parameters [Fig. 2(b), left] characterizing signal from voxels of noise and biofilm pores. The latter denotes voids or channels filled with optically transparent, low-scattering fluids that serve as distribution networks for feeding expanding MRSA colonies. Noise was filtered out at $\left[0<\sigma_{R}<9.9;R^{2}>0.98\right]$, and pores detected at $\left[10<\sigma_{R}<70;0.9<R^{2}<0.98\right]$. Fitting voxels with normal distribution established a range of parameters $\left[390<\mu <1750;320<\sigma_{N}<750;R^{2}>0.85\right]$ corresponding to metal hardware [Fig. 2(b), middle].

**Fig. 3:**
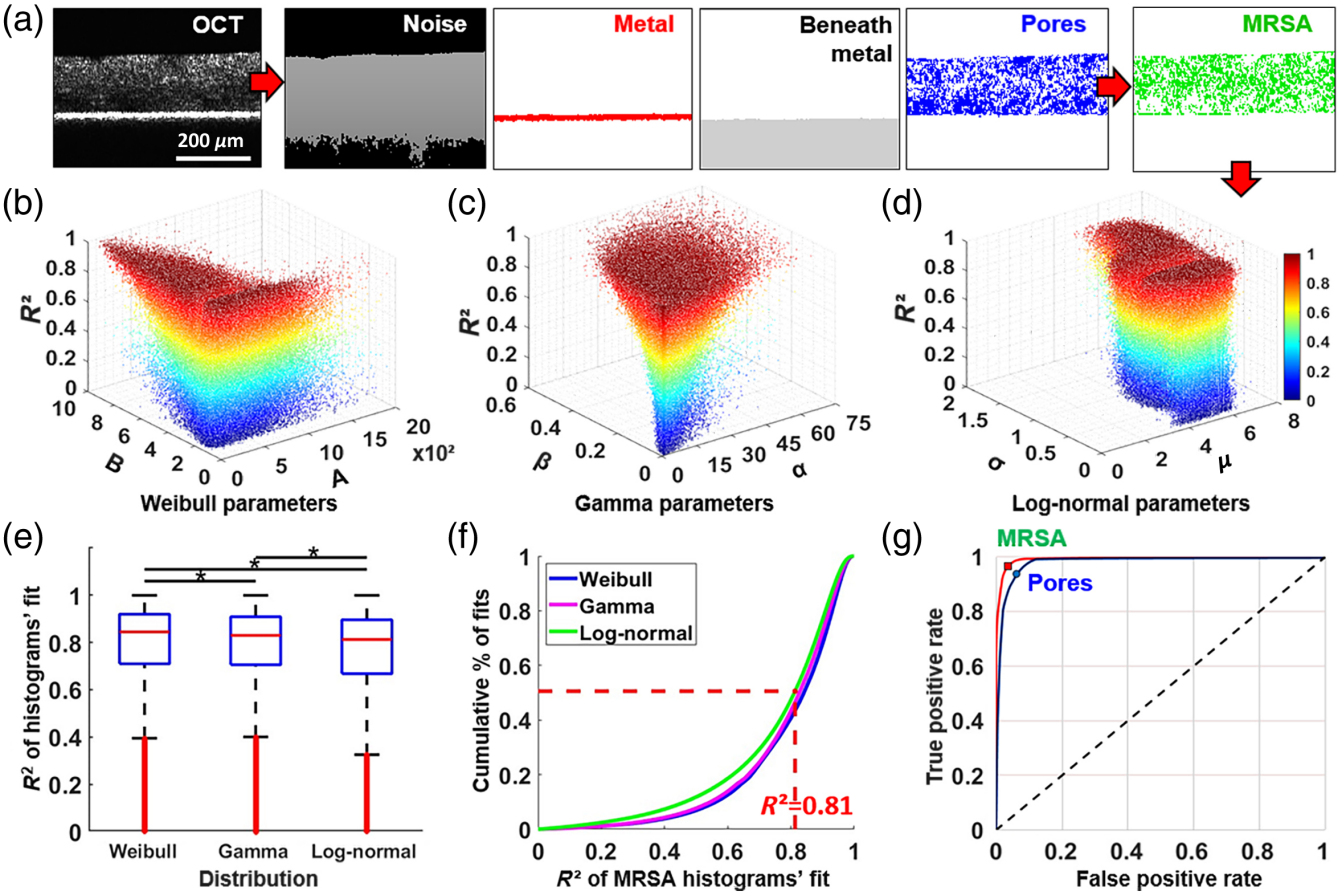
MRSA optical signature characterization. (a) Reference structural B-scan followed by detection and removal of noise (black), metal and beneath (red and gray), and pores (blue) in parametric images, effectively isolating subVOIs corresponding to MRSA clusters (green); (b)–(d) MRSA optical signature revealed as space of estimated parameters in fitting MRSA subVOIs with W, G, and LN distributions, presented in 3D with color gradation by $R^{2}$; (e) statistical significance (*p<0.05) found between mean $R^{2}$ obtained by each of three MRSA-fitting distributions; (f) cumulative percent of distribution fits where $R^{2}>0.81$ was determined as threshold for MRSA detection; (g) ROC curves for assessment of detection accuracy for MRSA and pores.

To isolate MRSA voxels in parametric images for characterization as in Fig. 2(b) (right), a masking procedure was followed as shown in Fig. 3(a). Noise (black), metal (red), below-metal (gray), and pores (blue) voxels were identified and filtered out in OCT images. In this controlled *in vitro* experiment, we assumed that the remaining voxels (green) corresponded to MRSA-related components of the biofilm microenvironment such as live/dead cells, extra-cellular polymeric substance (EPS) matrix, and pore smaller in size than the OCT resolution.

## 3 Results and Discussion

In this novel MRSA-OCT-texture-analysis approach, we investigated the performance of a combination of W, G, and LN, introducing the MRSA voxel fit parameters with $R^{2}$ for each distribution as shown in Figs. 3(b)–3(d). Analysis of $R^{2}$ among W, G, and LN fits using one-way ANOVA, then Tukey’s test, demonstrated significant differences in mean and variance of fit performance overall subVOIs [Fig. 3(e)]. The majority of MRSA subVOI histograms fit with $R^{2}$ between 0.80 and 1 [Fig. 3(f)]. Using this, we finalized a set of criteria for subVOI classification in OCT images: to be classified as MRSA, a subVOI histogram fit parameters must be localized into W, G, and LN parameter spaces shown in Figs. 3(b)–3(d), with goodness-of-fit $R^{2}>0.81$ (50th percentile). Essentially, clouds of tens of millions of fitting parameter data points, collected from OCT images of different MRSA biofilms, represent the unique optical signature of MRSA. Thus, the identification of key fitting parameters, corresponding to biofilm components (MRSA and pores), noise, and metal hardware, allowed for their delineation and color-coded visualization as shown in Figs. 2(c) and 2(d). The conclusion of this automated segmentation process is the computation of the following biofilm metrics:^34^ average thickness (mean of vertical sums of pixels that are not classified as noise or washer), relative roughness (average biofilm height deviation from, and normalized to, the mean thickness), and porosity (percent of biofilm volume occupied by pores).

Following delineation, we quantified the accuracy and discriminatory power of our method by analyzing the area under the receiver operating characteristic curve (AUC-ROC).^35^ A hundred locations of MRSA and pores in each of n=9 randomly selected 2D parametric images and corresponding OCT B-scans of biofilms were compared by two volunteers, focusing on areas of low signal in biofilms representing pores, and bright pixels—highly-scattering MRSA. ROC curves were produced as true-positives (sensitivity) versus false-positives (1-specificity) with the accuracy of detection ${\mathrm{AUC}}_{\mathrm{MRSA}}=0.9947$ and ${\mathrm{AUC}}_{\mathrm{PORES}}=0.9805$ [Fig. 3(g)].

**Fig. 4:**
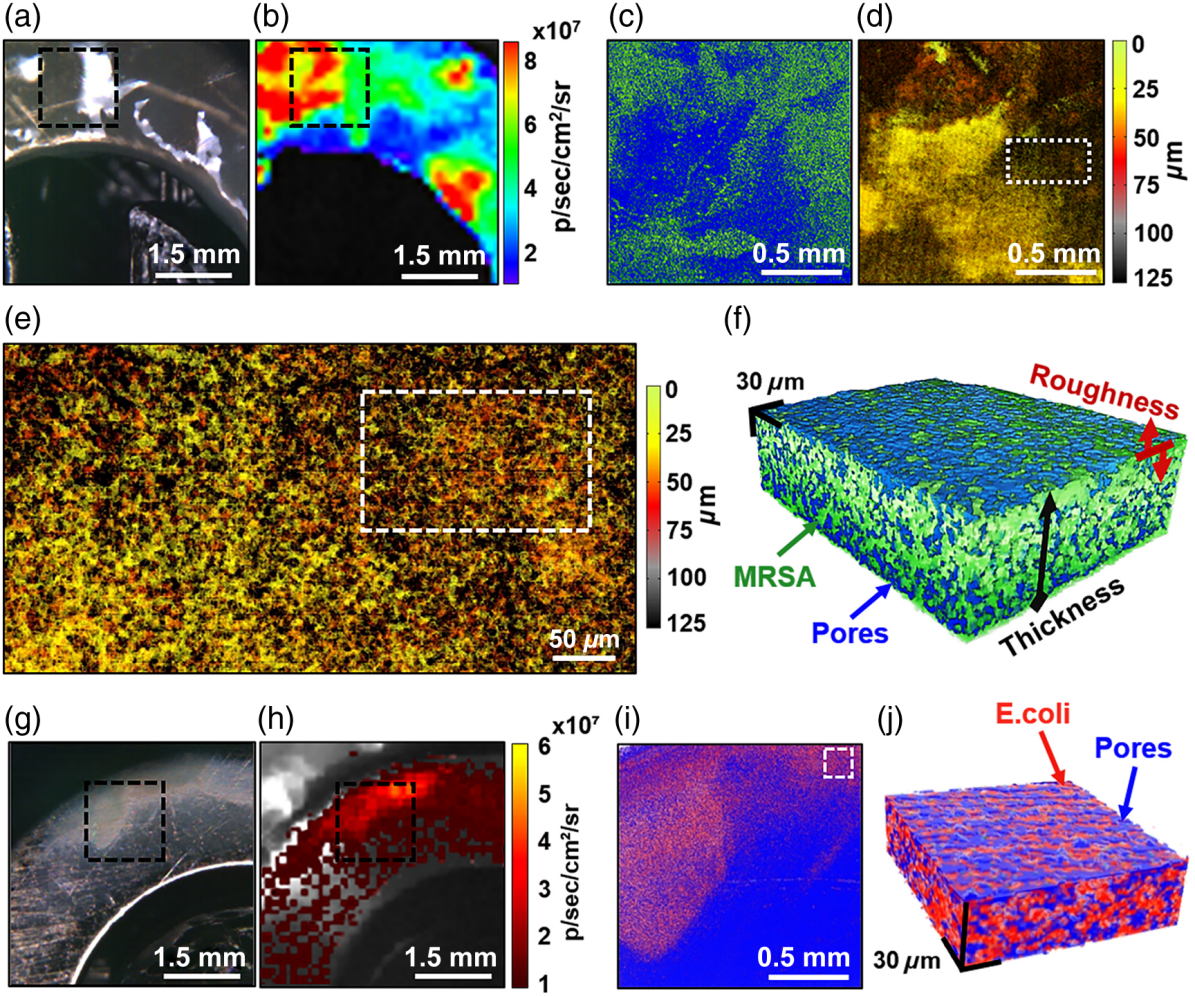
MRSA and *E. coli* biofilm and pores visualization and quantification. (a) Microphotograph and (b) luminescence of a MRSA biofilm grown on metal within 48 h; (c) combined parametric image of pores (blue) and MRSA (green), obtained from a 3D OCT image of the biofilm labeled with black-dashed rectangles in panels (a) and (b); (d) depth-encoded image of pores with color ranging from green (top layers) to gray (closest to metal surface); (e) enlarged section labeled with a white-dotted rectangle in panel (d), showing complex network of pores within the biofilm; (f) 3D image of MRSA (green) and pores (blue) from the area labeled with a white-dashed rectangle in panel (e). (g) Microphotograph and (h) fluorescence of *E. coli* biofilm grown on metal within 48 h; (i) combined *en-face* parametric image of pores (blue) and detected *E. coli* (red), obtained from a 3D OCT image of biofilm taken in black-dashed-rectangle region in panels (g) and (h); (j) 3D-rendered subvolume of detected *E. coli* (red) and pores (blue) from the white-dash-labeled rectangle in panel (i).

The combination of macro- and meso-scale biofilm detection provides a rich framework for *in situ* MRSA biofilm structure and formation analysis, including visual appearance [Fig. 4(a)], metabolic activity [Fig. 4(b)], and spatial organization of MRSA [Fig. 4(c)]. Also highlighted is the opportunity to obtain two- and three-dimensional images of biofilm pores [Figs. 4(d)–4(f)] at unprecedented resolution noninvasively. This access to the pore network invites future analysis and quantification for the investigation of biofilm maturation and response to treatment. As a methodology application example, the porosity of a 48-h MRSA biofilm shown in Fig. 4 was found to be 53.2%, with 90.4±21.3-μm average thickness and 18.7% relative roughness.

In this study, we characterize the MRSA optical signature by combining W, G, and LN distribution fit parameters and *R*² in OCT image texture analysis. Although utilizing three signal-characterizing distributions is computationally expensive, we consider the following biophysical explanation behind the need for each one. Gamma distribution fit parameters were found in OCT and US studies to correlate with scatterer density and effective scatterer cross-section,^20^^,^^36^^,^^37^ contributing to image speckle formation. Gamma distribution therefore may accurately reflect regions filled exclusively with MRSA, based on works demonstrating similar signal statistics in OCT images of microspheres—morphologically similar to MRSA. As shown in the embedded image in Fig. 2(b) (the bottom right of W/G/L-N middle panel, indicated by the arrow), a rough transformation of the typical gamma curve to the left (in the presence of lower signal) is the LN distribution, which captures MRSA features with occurrence of biofilm pores smaller than OCT resolution. Shifting the fitting curve to the right—W distribution—quantifies MRSA voxels with high-scattering debris, dead cells, or EPS matrix components. The potential of segmenting these individual components will be the subject of a future investigation.

The OCT-based biofilm analysis approach allows for (1) delineation of MRSA despite different levels of spatial organization (bacteria grouped in large colonies versus spaced out in smaller volumes); (2) detection of metal hardware—an important feature for clinical translation where the metal surface of most orthopedic hardware is not flat; (3) identification of pores and, therefore, ability to evaluate the role of porosity—one of the critical biological metrics^38^ in relation to biofilm maturity and response to treatment; and (4) automated quantification of biofilm thickness and roughness.

To further validate the specificity of the MRSA optical signature, we grew *E. coli* biofilms and identified their distinct optical signature. For this, we followed the same procedure as for MRSA, shown in Fig. 3 above. After the isolation of tens of millions of *E. coli*-only voxels in parametric images from different washers, we recorded the clouds of their fitting parameters, localized in W, G, and LN parameter spaces with goodness-of-fit $R^{2}>0.81$. Figures 4(g)–4(j) show a typical *E. coli* biofilm grown within 48 h (for details, see the figure caption). Its porosity was found to be 57.1%, with 33.5±5.2-μm average thickness and 16.3% relative roughness.

**Fig. 5:**
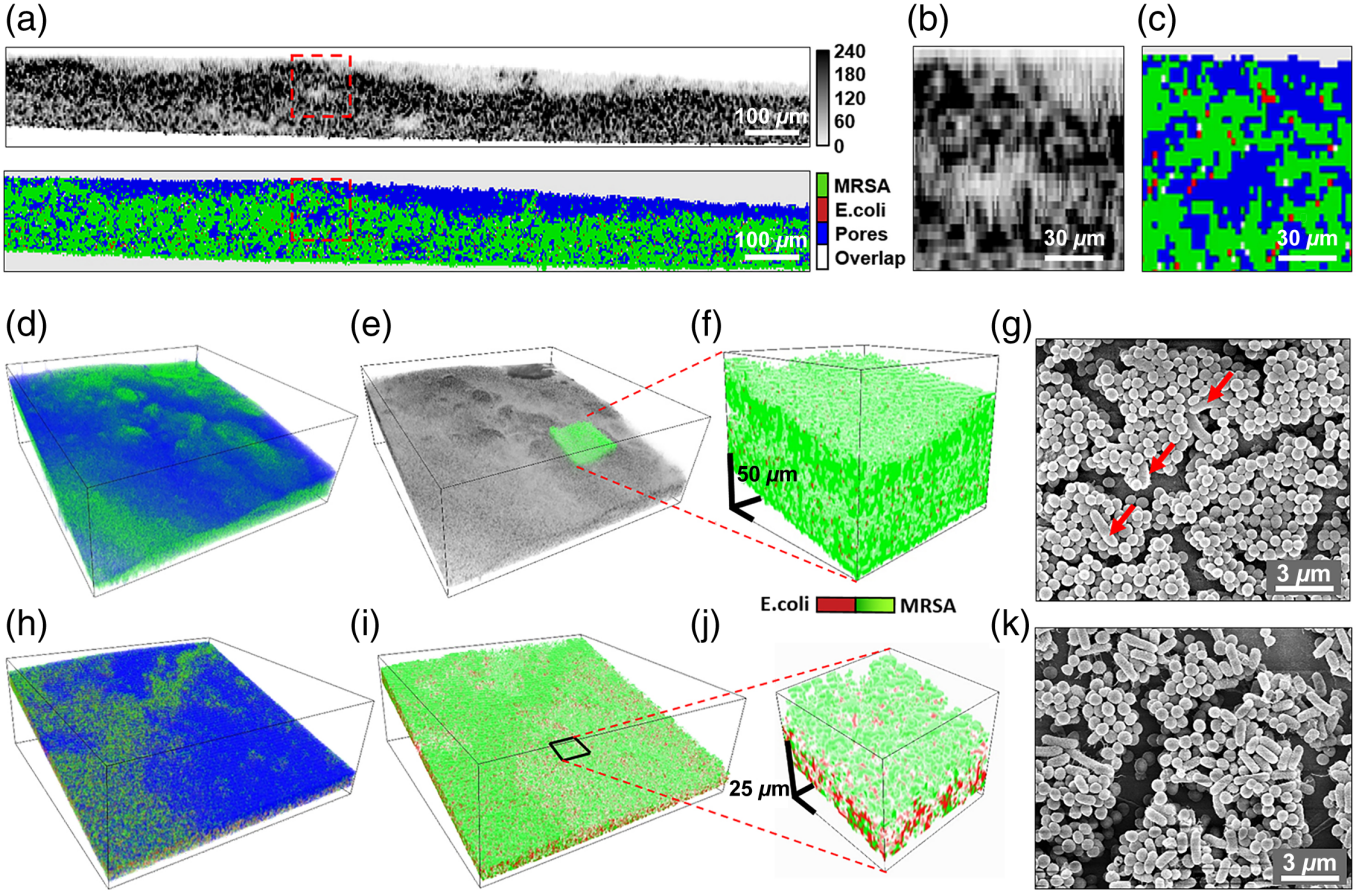
Detection of two bacteria types in MRSA*—E. coli* biofilms. (a) OCT cross-section of a 72-h old biofilm (top) and the corresponding parametric cross-section (bottom) with MRSA pixels in green, *E. coli*—in red, pores—in blue, and overlapping MRSA*–E. coli* pixels—in white colors; (b), (c) enlarged portions of OCT and parametric images, labeled with red dashed rectangles in panel (a). (d) 3D-rendered parametric image of the biofilm; (e) 3D-rendered OCT image of the same biofilm. The overlapping green structure indicates the location of (f) enlarged parametric subvolume, showing rare *E. coli* (dark red) bacteria inside the MRSA (green)-dominant biofilm; (g) SEM image of this biofilm, confirming the prevalence of MRSA with only a few *E. coli* cells (some labeled with red arrows) scattered across the MRSA clusters in the biofilm. (h) 3D-rendered parametric image of a 48-h old biofilm with MRSA (green), *E. coli* (red), and pores (blue); (i) the same biofilm but with pores removed; (j) enlarged parametric subvolume, showing that at earlier stages of biofilm development, there is more *E. coli* present; and (k) SEM image of this biofilm.

Under identical experimental conditions, we then grew MRSA—*E. coli* mixed biofilms and tested the developed parametric approach for delineation of these two bacterial species. One of the OCT cross-sections of such a biofilm is shown in Fig. 5(a) (top) with a corresponding parametric cross-section (bottom), with MRSA visualized in green color, *E. coli*—in dark red, biofilm pores in blue and pixels that were identified as belonging to either MRSA or *E. coli*—in white color. Enlarged portions of OCT and parametric images, labeled with red dashed rectangles in Fig. 5(a), are presented in Figs. 5(b) and 5(c), respectively, to demonstrate the distribution of these pixels. Although the OCT image visualizes dark bacteria clusters in panel (b), it is not necessarily clear where MRSA and *E. coli* are. Parametric analysis reveals which pixels belong to *E. coli* and which belong to MRSA and allows us to quantify their volumes within a biofilm. The whole 3D-rendered parametric volume is shown in Fig. 5(d), in which the dominating green color indicates a large prevalence of MRSA over *E. coli* in the mature biofilm.

The corresponding OCT image in Fig. 5(e) contains an embedded green parametric subvolume, enlarged in Fig. 5(f) to visualize how *E. coli* are scattered within dominating MRSA clusters. Indeed, SEM imaging of the same biofilm [Fig 5(g)] confirms that MRSA in this experiment at 72 h massively dominates *E. coli* with a manually calculated volume ratio of 96.5% MRSA: 3.5% *E. coli*. To compare these numbers with OCT, for this particular biofilm, we have found that MRSA occupied 49.5% of the total biofilm volume, *E. coli*—1.88%, and pores—47.8%. As preparation for SEM imaging includes a biofilm drying stage, obviously, quantification of pores is not possible using SEM, but at least, we can compare the MRSA–*E. coli* ratio, which with OCT imaging was found to be 96.3% MRSA: 3.65% *E. coli*. The small volume was identified as occupied by both bacteria [0.82%, white pixels in Fig. 5(c)]. This uncertainty, albeit small, originates from the limited resolution capability of the used OCT system. It indicates that within the OCT resolution volume, there may be a mixture of bacteria with close numbers of MRSA and *E. coli*.

In another example of the earlier stage of dual-species biofilm development, more *E. coli* was detected. Figure 5(h) shows a 3D-rendered parametric image of a 48-h old biofilm with 31.5% of MRSA (green), 24.4% of *E. coli* (red), 39.5% of pores (blue), 4.6% of both, MRSA, and *E. coli* classified voxels (white). When the pores are removed [Fig. 5(i)], one can see that MRSA tends to occupy more space on the biofilm surface, probably competing for access to nutrients. Below the surface, MRSA and *E. coli* ratios are comparable as can be seen from the enlarged portion in Fig. 5(j), with 52.6% of MRSA and 46.8% of *E. coli*. SEM image taken from the proximity of this location [Fig. 5(k)], reveals a 49.3% MRSA to 51.7% *E. coli* volume ratio. Because the primary goal of this study was not to explicitly analyze the biofilm parameters or the bacterial ratios at various stages of development, but rather to describe and validate the new methodology for their detection, the above numbers are provided for illustrative purposes, without further statistical evaluation.

The results presented here demonstrate that OCT is capable of distinguishing MRSA from other bacterial species *in vitro*, supporting the potential for clinical application in detecting MRSA on metal implants where polymicrobial biofilms are common. Traditional methods for detecting MRSA and other infections, such as culture-based assays and PCR, often take several hours to days, which may delay timely interventions and increase the risk of treatment failure. By contrast, noninvasive, portable, and high-resolution OCT imaging offers several advantages, including rapid detection and enhanced sensitivity to biofilm formation. In addition, the use of the developed biofilm pore imaging technique represents a breakthrough in the ability to visualize MRSA biofilms at an unprecedented level of detail. This method could allow clinicians to monitor biofilm formation in real time, assess the effectiveness of antibiotic treatments, and potentially identify early-stage infections that would otherwise remain undetected by traditional methods. As biofilm-related infections are a major concern in healthcare settings, the ability to reliably and rapidly identify MRSA in biofilms could revolutionize clinical diagnostic practices, enabling more effective and targeted treatment strategies. The proposed method could facilitate personalized medicine by enabling clinicians to tailor treatment options based on the specific characteristics of MRSA biofilms observed in individual patients. With the increasing prevalence of antibiotic resistance and the growing threat posed by MRSA in hospital settings, early and accurate detection is critical for improving patient outcomes and reducing healthcare-associated infections. Future clinical validation and refinement of the detection method, including its application in diverse clinical scenarios, would further underscore its potential as a transformative diagnostic tool in the fight against MRSA.

Translating this technique into clinical practice, however, would require addressing several key factors, as we have recently demonstrated in a pilot clinical study, involving five patients.^39^ To seamlessly integrate into the sterile surgical environment, a portable OCT probe would be required—one that can be easily maneuvered by the surgeon while being noninvasive and capable of real-time imaging. As surgeons need immediate feedback when detecting biofilms, particularly in complex cases where quick decision-making is critical, our methodology would need to provide high-resolution images rapidly through automated analysis algorithms, allowing for the identification and quantification of biofilm characteristics such as thickness, porosity, and spatial extent within seconds to minutes. Moreover, the method’s ability to detect biofilm formation on metal surfaces is particularly valuable in orthopedic surgery, where many implants are metal-based.^40^ A clinical implementation of this technology would need to accurately handle the varying geometries of metal surfaces, which is a crucial step for ensuring precise biofilm detection on implants. As demonstrated above using *E. coli*, beyond MRSA biofilms, the OCT-based method could be expanded to detect other bacterial strains commonly involved in orthopedic infections. Because bacterial strains can exhibit distinct optical signatures, further studies could refine and validate the use of OCT for detecting a broader range of infections, enabling its applicability in various surgical specialties, including neurosurgery and cardiovascular surgery, where implant-associated biofilms also present significant challenges.^41^

Although the current study provides valuable insights into the application of OCT for detecting and characterizing MRSA biofilms, several limitations related to the controlled nature of the experimental setup should be considered. First, the study focuses on two bacterial species (MRSA and *E. coli*), which could limit the generalizability of the findings to other pathogens typically encountered in clinical infections.^42^ Moreover, in a mixed microbial environment, there is the potential for overlap in optical signatures from different bacterial species within the OCT resolution volume as we highlighted in Fig. 5(c) with white pixels. If cells of different types are within the same resolution volume, texture analysis might identify a composite of their features,^43^ which could complicate the accuracy of distinguishing between species. This limitation could potentially affect the clarity and specificity of OCT imaging in real-world clinical applications, where multiple bacterial species might be present simultaneously. Further, the use of flat washers, although made from materials commonly used in medical implants (titanium and stainless steel), restricts the ability to assess how biofilm formation might vary on nonflat surfaces.^44^ In addition, our study relied on a specific growth medium (soy broth with 5% fetal bovine serum), which may not fully replicate the diversity of microbial interactions found in clinical environments.^45^ In real-world settings, implants often face exposure to a wide range of microorganisms and complex conditions, including polymicrobial biofilms,^46^ which may influence biofilm formation in ways not captured here. Although our inclusion of *E. coli* helps to establish the robustness of the OCT method for distinguishing MRSA in the presence of other bacteria, the study’s controlled conditions may not fully reflect the competitiveness of different species and the multifactorial nature of biofilm formation.^47^^,^^48^ Future studies will need to explore these complexities to further validate the clinical potential of OCT for biofilm detection.

## 4 Conclusion

This study introduces an OCT-based method for detecting and characterizing MRSA biofilms on orthopedic implants. Using distribution fit models, we successfully delineated MRSA from other biofilm components, quantifying metrics such as thickness, roughness, and porosity. The method also proved effective in distinguishing MRSA from other bacterial species, such as *E. coli*, in mixed biofilms, even on metal surfaces. OCT’s ability to visualize biofilm pores at unprecedented resolution offers new insights into biofilm development and treatment response. This noninvasive, real-time imaging technique has the potential to revolutionize MRSA detection and lead to more effective, personalized treatment strategies in clinical settings. Future studies will explore the complexities of polymicrobial environments, real-world clinical conditions, and the application of this method across various bacterial species and implant surfaces.

## Data Availability

The datasets generated and analyzed during the current study are not publicly available but may be obtained from the corresponding author on reasonable request.
